# Iodine-125 seed brachytherapy for early stage prostate cancer: a single-institution review

**DOI:** 10.1186/s13014-015-0349-0

**Published:** 2015-02-22

**Authors:** Simon Zuber, Susan Weiß, Dieter Baaske, Michael Schöpe, Simon Stevens, Stephan Bodis, Daniel R Zwahlen

**Affiliations:** Klinikum Chemnitz gGmbH, Klinik für Radio-Onkologie, Flemmingstrasse 2, D-09116 Chemnitz, Germany; The London Clinic, 22 Devonshire Place, London, W1G 6JA UK; Kantonsspital Aarau AG, Tellstrasse 25/Postfach, CH-5001 Aaarau, Switzerland; Kantonsspital Graubünden, Loëstrasse 170, CH-7000 Chur, Switzerland

**Keywords:** LDR brachytherapy, Dosimetry, Quality of life, Toxicity, Biochemical failure, Learning curve

## Abstract

**Purpose:**

We are reporting the five-year biochemical control, toxicity profile and dosimetric parameters using iodine-125 low dose rate brachytherapy (BT) as monotherapy for early stage prostate cancer at a single institution.

**Material and methods:**

Between April 2006 and December 2010, 169 men with early stage prostate cancer were treated with BT. Biochemical failure was defined using the Phoenix definition (nadir + 2 ng/mL). Treatment-related morbidities, including urinary, rectal and sexual function, were measured, applying the International Prostate Symptom Score (IPSS), the 7-grade Quality of Life Scale (QoL) and medical status, the International Consultation on Incontinence Modular Questionnaire (ICIQ), the International Index of Erectile Function (IIEF-5) and the Common Terminology Criteria for Adverse Events (CTCAE v4.03). Seed migration and loss, dosimetric parameters and learning effects were also analyzed.

**Results:**

Medium follow-up time was 50 months (range, 1–85 months). The five-year biochemical failure rate was 7%. Acute proctitis rates were 19% (grade 1) and 1% (grade 2), respectively. The overall incidence of incontinence was 19% (mild), 16% (moderate) and < 1% (severe). An increase in IPSS ≥ 5 points was detected in 59% of patients, with 38% regaining their baseline. Seed dislocation was found in 24% of patients and correlated with D90 and V100. A learning curve was found for seed migration, D90 and V100. QoL correlated with the general health condition of patient, incontinence symptoms and IPSS.

**Conclusions:**

BT for early stage prostate cancer offers excellent five-year biochemical control with low toxicities. QoL aspects are favorable. A learning curve was detected for procedural aspects but its impact on patient relevant endpoints remains inconclusive.

## Introduction

Prostate cancer is the most common malignancy in men in the developed world [1]. In Germany, the annual incidence is estimated to be as high as 48,650 new cases, accounting for approximately 22% of all cancer in males [2].

Iodine-125 low dose rate brachytherapy (BT) is an effective modality to administer a high dose to the prostate while minimizing toxicities for the adjacent organs at risk [3]. For selected patients with low and favorable intermediate-risk prostate cancer, BT is equally effective in controlling the disease as radical prostatectomy [4]. For men who place a premium on avoiding side effects of treatment and who accept the possible risk of late metastasis or death, also lack of treatment with an active surveillance strategy can be appropriate [5].

Judging the quality of an implant by dosimetric parameters is recommended by the American Brachytherapy Society (ABS) [6] and the European Society for Radiotherapy & Oncology, European Association of Urology and European Organization for Research and Treatment of Cancer (ESTRO/EAU/EORTC) [7]. Dose homogeneity within the target volume is much more difficult to achieve during BT than during external beam radiotherapy (EBRT), and implants are rarely geometrically perfect. To avoid partial underdosage of the target volume, it may be necessary to accept hot spots in other parts of the target volumes, but it is not clear how much dose heterogeneity should be considered excessive [8].

A known phenomenon after BT is the detachment of seeds from their initial site and migration to other organs. Seed migration may be identified in up to 55 percent of patients who are monitored with routine chest x-rays [9]. Also dislocation within the abdomen or pelvis is observed, with the majority of patients being asymptomatic [10]. Only very few migration-related sequelae have been reported, such as cases of myocardial infarction and radiation pneumonitis [11,12].

It stands to reason that quality of life (QoL) can be influenced by the adverse effects of BT. Typically, these are expressed in the gastrointestinal and genitourinary tract system. Toxicities after BT that have been reported are challenging to compare due to differences in follow-up time, disease profile at presentation, study endpoints and treatment regimens, which differ from a permanent implant alone to the combination with EBRT and/or androgen ablation [13]. According to the literature, urinary retention occurred in 5-22% of the patients after seed implantation alone [14,15]. Acute urinary symptoms increased by over 100% in patients treated either with BT alone or BT combined with EBRT. However, most studies found that about 90% of patients will have normalized their urinary complaints by one year postimplant [16,17]. Urinary incontinence after BT (without associated transurethral resection of the prostate) is reported in 0-19% of patients [18,19]. The incidence of radiation proctitis following implantation ranges from 1-19% in patients treated with implants alone [20,21]. Poor sexual function is reported in 43-48% of patients [22].

Several retrospective studies demonstrated the impact of implantation experience on the quality of the procedure: Lefur et al. [23], observed a learning curve to achieve consistent implantation parameters, which lead to a significant decrease in the dose to the rectum. They found no impact on the dosimetric parameters for the target volume, however. On the contrary, Hong-Wie Liu et al. [24] reported steadily decreasing numbers of patients with low D90s as their implant program matured. Keyes et al. [25] showed significantly falling acute urinary retention rates by order of implants. A learning curve could also be seen avoiding seed migration [26].

The aim of this study is to report our single-institution experience with BT for patients diagnosed with early-stage prostate cancer, highlight treatment related toxicities and analyze their relation to QoL.

### Patients and methods

All 169 men who were diagnosed with localized low- and intermediate risk prostate cancer and treated with BT as a monotherapy between April 2006 and December 2010 were enrolled in this retrospective study. Patient characteristics are shown in Table 1. No patients were excluded. BT was indicated according to above mentioned guidelines; intermediate-risk patients were considered for brachytherapy on a case-by-case basis. Monotherapy was used judiciously at the discretion of the treating physicians and upon the patients’ preferences. Median age and PSA value at diagnosis were 68.5 years (range: 47–79) and 7.2 ng/mL (range: 0.14-14.88), respectively. The majority of patients (91.8%) had a Gleason score of six (range: four to seven), while 1.2% had a Gleason score of seven. Treatment outcome was assessed using the Common Terminology Criteria for Adverse Events (CTCAE v4.03) for proctitis, the international prostate symptom score (IPSS) [27] and a questionnaire for prostate cancer patients developed by the German Cancer Society (DKG). Included in this questionnaire is the self-evaluation of QoL and medical condition using scales from zero to seven. These correspond to items used in validated questionnaires of the EORTC [28]. Further items are questions about urinary and rectal continence according to the international consultation on incontinence modular questionnaire (ICIQ) [29] and the abbreviated five-item version of the international index of erectile function (IIEF-5) [30]. Patients had a follow-up on a regular basis, four to six weeks, then three, six, 12, 18, 24 and 36 months post implantation. The medium follow-up time was 50 months (range: 1–85). All patients gave their informed consent regarding the use of their records for this study. The Klinikum Chemnitz data security officer approved the study.

**Table 1:** **Patients’ characteristics**

**Age (year)**		
	Mean ± SD	68.5 ± 5.6
PSA at diagnosis (ng/mL)		
	Mean ± SD	7.2 ± 2.6
	10 or less	144
	10-20	25
Clinical T stage		
	T1c	143
	T2a	16
	T2b	7
	T2c	1
	Ne	2
Gleason score		
	4-6	166
	7	2
	Ne	1
Neoadjuvant ADT		
	No	125
	Yes	42
	Ne	2
IPSS at baseline		
	Mean ± SD	6.3 ± 4.0 (*)
Follow-up period (months)		
	Mean (range)	50 (1–85 months)

SD: Standard deviation, Ne: Not evaluated.

ADT: Androgen deprivation therapy.

IPSS: International prostate symptom score.

(*) 162 patients included.

Treatments were performed with 125-Iodine seed BT by two experienced urologists using a Mick applicator. 160 Gy to the prostate was prescribed. Collected data included the prostatic volume, D90 (isodose enclosing 90% of the prostate in Gy and percent), V100 of the prostate (percentage volume of the target receiving 100% of the prescribed dose), rectal V100 (volume receiving 100% of the prescription dose) at implantation and after treatment, and urethral D30 (isodose enclosing 30% of the urethra), at implantation only. The dosimetric parameters are summarized in Table 2. We aimed for a V100 ≥ 95%, urethral D30 < 130%, and a rectal V100 < 1 cc (evaluated for 145 Gy). For orientation, values of V90.6 (equals volume receiving 145 Gy) were additionally recorded. Despite existing target values, patients whose parameters did not reach those at post-implant assessment did not receive any further treatment. Forty-two patients (24.4%) received neoadjuvant androgen deprivation therapy (NADT). Short-term anti-hormonal treatment was used for the purpose of prostate down sizing before brachytherapy and was based on implanting surgeons’ individual decisions. No threshold was defined. CT-based postimplant dosimetry was routinely performed 4–6 weeks after BT for all patients.

**Table 2:** **Dosimetric parameters**

	**No. of pts.**	**Mean ± SD**	**Min**	**Max**
Urethra D30 [Gy] at implantation (1)	167	185.5 ± 6.7	159.8	204.5
Prostate V [cc] at implantation (2)	150	31.9 ± 11.3	12.8	63.0
Prostate V [cc] postimplant (2)	163	31.8 ± 11.1	13.8	61.4
Prostate D90 [Gy] at implantation (3)	167	185.5 ± 4.0	173.7	198.4
Prostate D90 [Gy] postimplant (3)	165	179.2 ± 16.3	118.6	210.6
Prostate V100 [%] at implantation (4)	167	99.6 ± 0.5	97.5	100
Prostate V100 [%] postimplant (4)	165	94.9 ± 3.8	80.7	100
Prostate V90.6 [%] at implantation (5)	167	99.9 ± 0.2	98.4	100
Prostate V90.6 [%] postimplant (5)	165	97.0 ± 3.8	64.5	100
Rectum V100 [cc] at implantation (6)	167	0.3 ± 0.2	0	1.2
Rectum V100 [cc] postimplant (6)	165	0.5 ± 0.6	0	3.2

(1) dose to 30% of the urethra.

(2) volume of the prostate.

(3) dose to 90% of the prostate.

(4) percentage volume receiving 100% of the prescription dose.

(5) percentage volume receiving 90.6% of the prescription dose.

(6) volume of the rectum receiving 100% of the prescription dose.

Data collection, management, analysis and visualization were performed with Microsoft Office Professional 2007 and IBM SPSS Statistics for Macintosh, Version 22.0. Armonk, NY: IBM Corp. P-values below 0.05 were considered statistically significant.

In order to reach a higher data quantity for a valid interpretation, an additional written survey was conducted. Of all patients, 111 returned completed questionnaires. Self-estimated QoL was correlated with health status, ICIQ, IIEF-5, IPSS and GI toxicity using Spearman’s rho. Moreover, all potentially influencing dosimetric and pre-therapeutic parameters were correlated with these outcome variables, e.g. the urethral D30, volume of the prostate and pre-therapeutic IPSS with ICIQ scores or, the rectal V100 and volume of the prostate with GI toxicity. The strength of correlation was interpreted as weak for ρ < 0.5, moderate for 0.5 ≤ ρ < 0.8 and strong for ρ > 0.8. As for interpretation of the IPSS, we followed the definition of an absolute rise in score of five points to represent clinically significant worsening in the patients’ urinary symptoms, as initially used and validated by Cesaretti et al. [31].

To determine whether there were differences between the implant qualities of the two surgeons, an unpaired t-test was employed. The tested variables are metric and the sample size is large enough. According to the central limit theorem, it can thus be employed without tests on normal distribution. To assess the equality of variances for all variables calculated for the two groups, Levene’s test was used.

To investigate a possible pattern of learning effects, the study population was divided into a group of 85 patients (early implantations) and 84 patients (late implantations), respectively. As depicted above, Levene’s and unpaired t-tests could be used for comparison. Included variables were the number of lost or dislocated seeds and the above-mentioned dosimetric parameters. Data were also evaluated separately for both surgeons.

## Results

**Figure 1:**
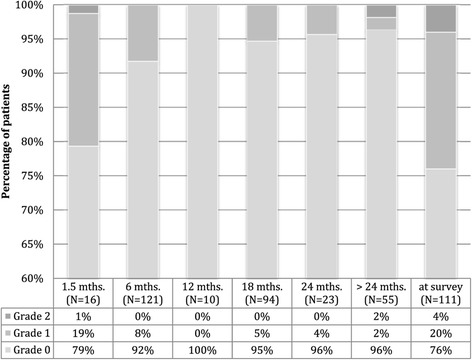
**Proctitis grading according to CTCAE v.4.03 (grade 0: no symptoms).**

The incidence of proctitis at different time points after BT is displayed in Figure 1. The number of patients available for analysis is provided in brackets. No grade three or higher events were reported.

For 105 men, grading of proctitis at 1.5 months after treatment was obtained, and at least another data point during further follow-up. Of these patients, 21% (n = 22) showed improvement of proctitis symptoms in follow-up. As many as 9.5% (n = 10) experienced worsening of associated complaints (nine patients with grade one and one patient with grade two morbidity).

**Figure 2:**
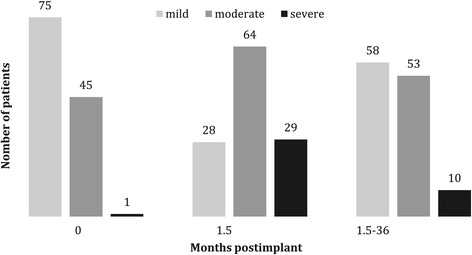
**Voiding symptoms according to International Prostate Symptom Score.**

The IPS-scoring system allows urinary symptoms to be classified as mild (IPSS 0–7), moderate (IPSS 8–19), or severe (IPSS ≥ 20). Included in this subanalysis were 118 patients who completed the IPSS questionnaire before BT, at first follow-up (1.5 months) and at least one more time between three months and three years post implantation. Figure 2 displays the prevalence of symptoms at different points in time after BT. Table 3 shows the number of patients evaluated with an increase of more than five points in post treatment IPSS at 1.5 months, and a decrease of ≥ 5 beyond 1.5 months, respectively.

**Table 3:** **Post-treatment changes in IPSS**

	**No. of pts. (%)**	**IPSS (mean ± SD)**
Included patients	118 (100%)	11.2 ± 5.1
≥ 5-point increase at 1.5 months	70 (59%)	6.8 ± 3,3
≥ 5-point decrease after 1.5 months	45 (38%)	4.6 ± 2.1

IPSS: International prostate symptom score, SD: Standard deviation.

**Figure 3:**
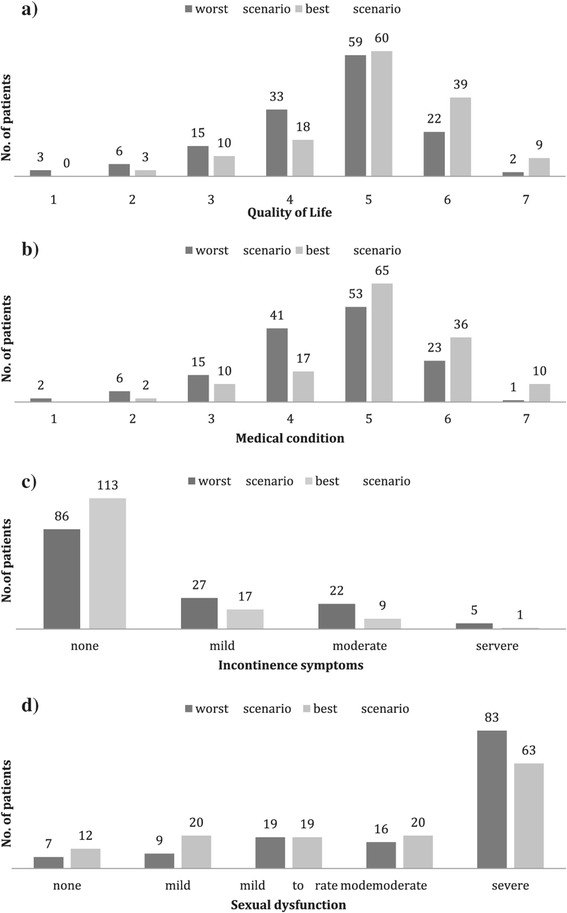
**Treatment outcome. a**. Quality of Life (7-grade scale). **b**. Medical condition (7-grade scale). **c**. Incontinence symptoms according to International Consultation on Incontinence Questionnaire. **d**. Sexual dysfunction according to International Index of Erectile Function (5-item version).

A total of 135 patients completed at least one DKG questionnaire. Six patients completed all subcategories except the IIEF-5. Forty-three patients completed one, 81 patients two and 11 patients three questionnaires. When more than one questionnaire per patient was obtained, the worst and best results within the follow-up period were utilized. The results of this self-estimation and distribution after BT are illustrated in Figures 3a-d. The 7-item scales for QoL and medical condition were construed as follows: 1 very discontent, 2 discontent, 3 rather discontent, 4 neither/nor, 5 rather content, 6 content, 7 very content.

**Figure 4:**
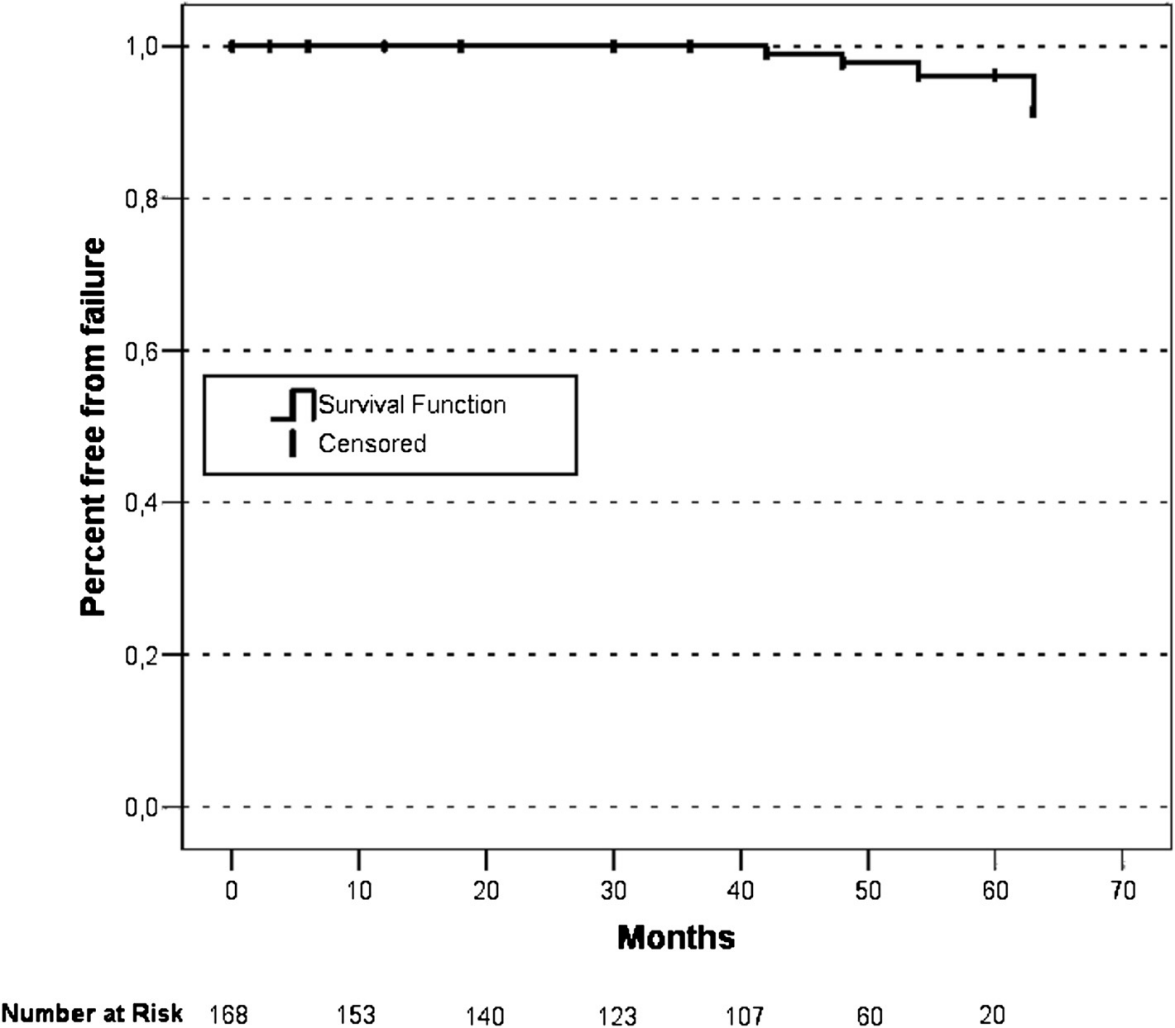
**Kaplan-Meier estimates.**

Biochemical failure was defined as a rise of two ng/mL or more above nadir, and based on the RTOG-ASTRO Phoenix Definition [32]. For calculation of the five-year biochemical free-survival, 59 patients with a follow-up of at least 60 months or prior recurrent disease were included. One pretreatment and at least two PSA values after BT were available. Table 4 shows the further details. Four patients (7%) showed PSA failure in accordance with the RTOG-ASTRO Phoenix definition, occurring at 42, 48, 54, and 63 months, respectively. Figure 4 shows the Kaplan-Meier estimates.

**Table 4:** **PSA course**

**PSA**	**Evaluable pts.**	**Pts. with PSA failure**	**Pts. with a follow-up more than 60 months**
No. of pts. (%)	141	6 (4.3%)	55 (39.0%)
Pretreatment PSA	7.1 ± 2.5	6.3 ± 1.5	7.0 ± 2.5
PSA Nadir	0.4 ± 0.6	1.3 ± 1.6	0.1 ± 0.2

SD: Standard deviation.

For a total of 164 patients, we were able to investigate the pattern of seed migration or loss. Most patients, n = 127 (75.6%), did not experience seed loss. The dislocation of a single seed was observed in 29 (17.7%) patients. Two seeds were lost in eight (4.9%), three in two (1.2%), and four in one of the patients. Predominant sites of migration/dislocation were to seminal vesicles, periprostatic tissues and lungs, occurring in 27, 13, and 10 patients, respectively. For one patient, a single seed that was lost could not be identified. Another patient lost a seed during urination. Furthermore, single seed embolization into the spinal canal, presacral space, and a deferent duct was observed in three patients (one patient for each site).

Almost all QoL-related aspects (7-grade scales and genitourinary symptoms) that were obtained by questionnaires or upon consultation within the observation period showed a positive correlation at different points in time, except for proctitis. To determine the association of these aspects, pre-implant and dosimetric parameters, the last retrieved questionnaires were used. Dependent variables are shown in Table 5. Represented are those correlation coefficients where statistical significance was given.

**Table 5:** **Significant correlations**

	**No. of pts.**	**Spearmen-Rho**	**p-value**
Quality of life			
Health status	111	0.882	0.000
ICIQ (1)	111	−0.211	0.027
IPSS (2)	104	−0.204	0.037
Urethral D30 (3)			
Volume of prostate	162	−0.326	0.000
IPSS at survey			
IPSS (preimplant)	103	0.287	0.003
Seed dislocation/loss			
V100 (4)	163	−0.262	0.001
D90 (5)	163	−0.190	0.015

(1) ICIQ: international consultation on incontinence modular questionnaire.

(2) IPSS: international prostate symptom score.

(3) D30: dose to 30% of the urethra.

(4) V100: volume of the prostate receiving 100% of the prescription dose.

(5) D90: dose to 90% of the prostate.

## Discussion

Due to the similar size of the referral base, many institutions in Germany as well as in other European countries offering BT have a comparable patient throughput. It is still important that patient series from smaller departments are analyzed to increase the quantity of comparable results, particularly since there are no randomized controlled studies to date that would permit an evidence-based assessment of the benefit of BT over other treatment options. In Germany, directives for the benefit catalogue of the statutory health insurance funds are issued by the G-BA (Federal Joint Board): Accordingly, there is no evidence regarding the patient-relevant endpoints, such as overall survival and diseases specific mortality. However, it has been stated that there is “some” evidence in terms of treatment toxicity: Rectal function may be more impaired when compared to EBRT, while sexual dysfunction and urinary incontinence may be less when compared to prostatectomy [33]. A decision process regarding general coverage of BT has been suspended, providing that higher evidence by adequate studies is achieved. During the method evaluation process, BT is normally only funded by statutory insurances in a hospital setting. Exploitable results of larger prospective randomized trials, such as the German prostatic cancer study PREFERE [34], are expected.

Overall disease free survival at five years in patients after BT differ between 76-93% [35-37]. As with toxicity, the series with BT as a monotherapy are difficult to compare due to differences in the follow-up time and treatment regimens. Furthermore, divergent definitions for biochemical failure were chosen. In this study, the rate of biochemical failure according to the RTOG-ASTRO Phoenix Definition [32] was low. The five-year biochemical recurrence-free survival was 93% for patients with a follow up of at least 60 months or prior recurrent disease. The actual rate may be even higher. Many patients had low PSA-values upon follow-up but could not be included in the subgroup analysis, due to lacking further values. Despite the relatively small number of patients included, our results are encouraging and in line with results from above cited studies.

Figures 1, 2, 3 demonstrate a good outcome in our study population in terms of aspects related to QoL, except for sexual dysfunction. Voiding symptoms decreased over time. There was also a low rate of proctitis according to toxicity grading, and the majority of patients had no signs of urinary incontinence. Most patients stated being rather content, content or very content with QoL. Self-appraisal of QoL was associated most strongly with that of general health condition (ρ = 0.8) and weakly correlated with the ICIQ (ρ = −0.21) and IPSS (ρ = −0,20). There was no significant correlation between QoL, the rate of proctitis or the IIEF-5 score.

From experience, one of the reasons men seek implantation over surgery when considering their treatment options for early-stage prostate cancer is the potentially lower risk for ED. In this study, ED was assessed with the IIEF-5. ED was not associated with worsening of QoL. Explanations for this finding might include a declining sexual interest with age (mean: 68.5 years in this study) or acceptance of sexual dysfunction over time. Furthermore, the IIEF was not designed to assess the impact of ED on psychosocial outcomes that influence sexual function. In fact, it was developed primarily for use as an efficacy measure in clinical trials of ED [38]. Its 5-item version is a simple office screening instrument to diagnose the presence and severity, with a focus only on erectile function and intercourse satisfaction [30]. Moreover, male sexual dysfunction includes diminished libido or abnormal ejaculation, issues that are not addressed in the questionnaire. Various conditions are known to be associated with ED, such as cardiovascular disease, obesity, diabetes mellitus, certain drugs, or depression [39]. In this study, patients with comorbidities were not excluded. Though representative for the general population, our data would not allow to clearly attribute the pattern of ED to BT.

Due to the anatomic proximity of the prostate to the urethra and bladder, both acute and late toxicity can be observed. Most patients develop an acute worsening of urinary symptoms, such as increased frequency, urgency, nocturia, etc. These symptoms resolve in most cases [40]. We assessed urinary symptoms using the IPSS and observed an increase of ≥ five points in 59% of the patients, approximately six weeks post implant. Of these, 38% reached their baseline level. In a study by Keyes et al., similar results were found in 712 men with localized prostate cancer treated with an I-125 implant between 1998 and 2003 [40]: By six weeks after treatment, most patients experienced an acute exacerbation of urinary symptoms, which was associated with an increase in the IPSS to a mean of approximately 19. Symptoms resolved within six months in 65% of cases and within one year in 91%. Associated factors included more baseline urinary symptoms and more intense symptoms in the immediate post-treatment period. In our patients, there was a weak correlation between the last retrieved IPSS and the pretherapeutic scores. Neither the size of the prostate nor the urethral D30 impacted the IPSS. Serious rectal injuries (fistula) are rare [41] and were not observed in our study. An accurate implant technique can minimize the radiation dose to the anterior rectal wall and brachytherapy-related bowel morbidity. This was shown, for instance, by Snyder et al. [42], who demonstrated a correlation between the V100 and the development of grade 2 proctitis: If ≤ 0.8 cc of rectal wall received 160 Gy, no proctitis developed. The likelihood increased to 5% in case ≤ 1.3 cc received 160 Gy. In our study, the incidence of proctitis complaints was low. There was no significant association with QoL and the incidence was not influenced by rectal dosimetric parameters.

After BT, loose seeds are particularly prone to loss and movement. The loss of multiple seeds can cause a substantial reduction of D90 coverage. Stranded seeds are less prone to be lost [43]. A weak but statistically significant correlation between the number of lost seeds and dosimetric parameters (V100, D90) could also be shown in this study. The clinical implications of this are unclear. A prescription dose of 160 Gy to the prostate used to be the local standard during the first years of implantations, leaning upon a dose–response analysis by Stock et al. [44]: They recommended a range of 140–160 Gy delivered to the prostate as defined by the D90. In practice however, many brachytherapists plan a higher dose [6] to compensate for edema or seed placement uncertainty and D90s from 180 to 200 Gy seem to be well tolerated with low risk of toxicity [45]. As for the urethra, one may aim for an UV30 < 125% [6] or a D30 < 130% of the prescription dose when referred to GEC-ESTRO recommendations [7]. At the rectum, the RV100 is ideally < 1 cc at day 1 dosimetry and < 1.3 cc at day 30 (due to the resolution of periprostatic edema and, thus, changing proximity) [6].

A thorough evaluation of differences between day 0 and post-implant dosimetry was beyond the scope of this work. Prostate edema after BT is well recognized and can result in a decrease in dose coverage [46]. The extent and time course of prostate edema and its effect on dosimetry have been analyzed for example by Taussky et al. [46] In this study, we compared the dosimetric results of two experienced surgeons. Our analyses indicate a significant difference between the surgeons for urethral D30. However, both reached values within recommended constraints. For other planning parameters, for neither seed loss nor dislocation, no significant differences were found. Moreover, we looked at two different time periods to evaluate possible learning effects as described in the literature. Our observation of a learning curve for variables defining the quality of an implantation partially corroborates effects that were previously noted: Within our chosen observation periods, significant changes were demonstrated: Less seeds were lost or dislocated and values of V100 and D90 increased over time. A closer examination performed separately for the implanting physicians reveals a slightly different picture. Both surgeons reached a significantly improved rate of seed loss over time, whereas a significant increase of V100 was only observed for one. Despite these effects, the clinical relevance remains unknown. In terms of tumor control probability, the D90 is particularly important [44]. Moreover, the V100 could be a good clinical indicator for a successful implant. As for D90, obtained values for the early 85 implants was 174.6 Gy and, 184.0 Gy for the succeeding 84 implants: again, both meeting the recommendations. Recording all parameters separately for different implantation teams served as an additional means of quality control. Though statistically significant, the differences of the implant quality were minor. As a matter of fact, a higher learning capacity or better manual skills could not be derived from our data. In most prostate LDR brachytherapy programs, it is seen that the implant quality increases with the number of procedures performed up to about 70 to 100 procedures [47,48]. However, technological advances have taken place and made the implantation procedure more precise [48].

## Conclusion

BT constitutes one of many effective and safe treatment options for early-stage prostate cancer. The five-year biochemical relapse-free survival is excellent and treatment toxicity is favorable. However, treatment-related side effects, even when mild, have to be discussed with the patient. Quality of life and its related aspects are generally good. Learning effects by number of implants occur, but their impact on patient-relevant endpoints is inconclusive from our data.
